# Development of a Systematic Review Protocol and a Scoping Review of Ultrasound-Induced Immune Effects in Peripheral Tumors

**DOI:** 10.1007/s11307-021-01686-x

**Published:** 2021-11-29

**Authors:** Anne Rix, Renée Girbig, Céline Porte, Wiltrud Lederle, Cathalijn Leenaars, Fabian Kiessling

**Affiliations:** 1 Institute for Experimental Molecular Imaging, Medical Faculty, RWTH Aachen International University, Aachen, Germany; 2 Department for Health Evidence, Radboud Institute for Health Sciences, Radboud University Medical Centre, 6525 GA Nijmegen, The Netherlands; 3 Department of Population Health Science, Unit Animals in Science and Society, Utrecht University, 3508 TD Utrecht, The Netherlands; 4 Institute for Laboratory Animal Science, Hannover Medical School, 30625 Hannover, Germany

**Keywords:** Therapeutic ultrasound, Diagnostic ultrasound, Microbubbles, CEUS, Immune system, Systematic review protocol, Scoping review

## Abstract

**Purpose:**

Publication numbers reporting that ultrasound can stimulate immune reactions in tumors steadily increase. However, the presented data are partially conflicting, and mechanisms are difficult to identify from single publications. These shortcomings can be addressed by a systematic review and meta-analysis of current literature. As a first step, we here present the methodology and protocol for a systematic review to answer the following research question: Does ultrasound alter the immune reaction of peripheral solid tumors in humans and animals compared to control conditions without ultrasound?

**Procedures:**

We designed a protocol to perform a systematic review and meta-analysis. The suitability of the protocol to detect and sort relevant literature was tested using a subset of publications. We extracted study characteristics, ultrasound parameters, and study outcomes to pre-evaluate the differences between publications and present the data as a scoping review.

**Results:**

From 6532 publications detected by our preliminary literature search, 320 were selected for testing our systematic review protocol. Of the latter, 15 publications were eligible for data extraction. There, we found large differences between study characteristics (e.g., tumor type, age) and ultrasound settings (e.g., wavelength 0.5–9.5 MHz, acoustic pressure 0.0001–15,000 W/cm^2^). Finally, study outcomes included reports on cells of the innate (e.g., dendritic cells, macrophages) and adaptive immune system (e.g., CD8-/CD4-positive T cells).

**Conclusion:**

We designed a protocol to identify relevant literature and perform a systematic review and meta-analysis. The differences between extracted features between publications show the necessity for a comprehensive search and selection strategy in the systematic review to get a complete overview of the literature. Meta-analyses of the extracted outcomes can then enable evidence-based conclusions.

**Supplementary Information:**

The online version contains supplementary material available at 10.1007/s11307-021-01686-x.

## Introduction

**Fig. 1.**
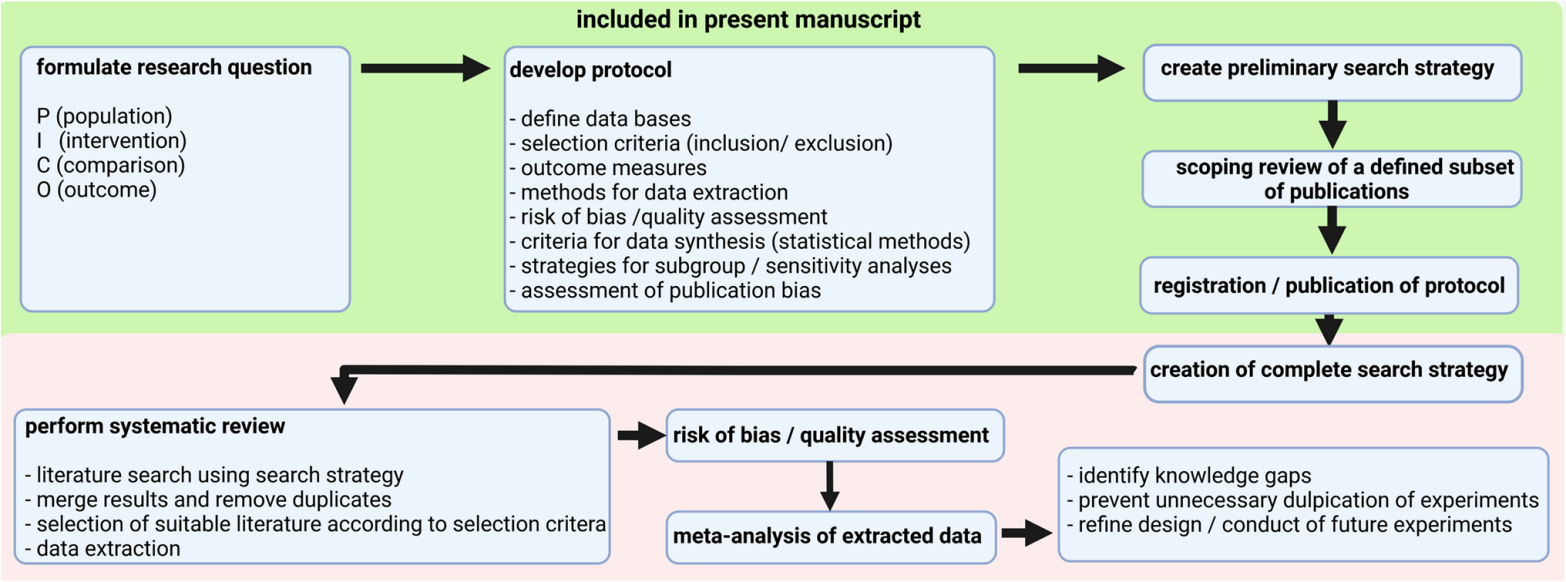
Flowchart of our systematic review. The conduct of a systematic review is based on a structured methodology. This manuscript aims to publish the research question, protocol, a preliminary search string, and a scoping review of a defined subset of publications. The systematic search, data extraction, and meta-analysis will be performed after the publication of the protocol (created with BioRender.com).

The commonly known narrative reviews provide a descriptive overview of the current literature, such as published by Wu et al. [1], van den Bijgaart et al. [2], or Joiner et al. [3] on the topic of ultrasound-induced immune effects. They often consist of publications known to the author and thus descriptively summarize only a subset of the literature. Therefore, narrative reviews always run the risk of being influenced by the opinion of a single researcher [4]. In contrast, systematic reviews and meta-analyses aim to generate new results from the analysis of all relevant literature about a particular research question, using pre-defined systematic and transparent methods specified in a protocol. These reviews are characterized by a comprehensive systematic search, validity assessment, systematic presentation of the results of included studies, and systematic synthesis of evidence (ideally using meta-analyses) [5]. It is important to emphasize that data are extracted from included publications and combined in meta-analyses to draw new scientific conclusions. The first step in a systematic review is the definition of a specific research question using the PICO format (population, intervention, comparison, outcome). Then, a tailored protocol is developed to answer that specific question. It includes selection criteria for publications, defines outcomes, and describes the specific methodology for data extraction and analysis, as well as the strategies for assessing the quality of the included publications. In addition, the National Institute for Health Research (NIHR) recommends the registration of protocols for systematic reviews in publicly available databases [6], similar to the mandatory registration of clinical trials in public registries. An additional publication of protocols in peer-reviewed journals is also common [7–10]. Protocol registration and publication aim to increase the transparency of methods [4], reduce the risk of subsequent protocol adjustment to results (e.g., requesting publication of all pre-specified results regardless of outcome), and minimize potential duplication. Therefore, strict application of a pre-defined protocol provides more reliable results. The next step is to develop a search strategy that covers the four topics (PICO) of the research question and systematically identifies all available publications. These are screened in two selection phases to identify all publications eligible for extraction of the pre-defined study characteristics and outcomes. The results are then compared and analyzed by meta-analyses using specific statistical methods, which even allow the comparison of effect sizes between studies with large differences in their study design. Subsequent subgroup analysis helps identify potential sources influencing the effect [11]. Finally, the distribution of all effect sizes in a funnel plot can be used to identify the occurrence and degree of small-study effects [12]. Furthermore, a risk of bias analysis can be performed to evaluate the study designs’ quality [13]. A flowchart of our systematic reviews can be found in Fig. 1. Comparing outcomes of different publications can be particularly challenging in preclinical research due to the large differences between and inadequate reporting of study designs, and the avoidance of publishing negative results can lead to publication bias [14].

One field of cancer research presenting a large difference between experiments is diagnostic and therapeutic ultrasound. Although ultrasound-induced effects on tumors are still a subject of ongoing research, especially the suitability of focused ultrasound as a tool to induce anti-tumorigenic immune responses and to enhance the efficacy of immunotherapy is currently under intensive investigation [1–3]. Depending on the applied frequency, pressure, pulse length, duty cycle, and treatment time, ultrasound produces predominantly thermal or mechanical effects in tissues. Applying long ultrasound pulses with high pressure leads to a rapid temperature increase to 60–85 °C causing tissue coagulation and cell necrosis [2]. Ultrasound protocols using lower pressure can be used to heat tissue to 40–45 °C to induce thermal stress (few minutes) or hyperthermia (up to 90 min), both leading to cell death [15]. Next to thermal effects, ultrasound can also mechanically interact with tissue, e.g., by applying short ultrasound pulses with high pressure to produce cavitating bubble clouds (cavitating cloud histotripsy) or boiling bubbles (boiling histotripsy), resulting in a tissue breakdown without considerable heating [16]. In contrast, lower acoustic intensities induce temporary mechanical stress with less apoptosis or necrosis. Ultrasound contrast agents (e.g., gas-filled microbubbles) can locally amplify mechanical or thermal ultrasound effects. In this regard, a stable oscillation of microbubbles induces moderate shear stress on endothelial cells due to acoustic microstreaming [17]. In contrast, inertial cavitation of microbubbles generates high temperatures or pressures, resulting in the generation of reactive oxygen species or microjets, leading to permanent damage of nearby cells [18].

Mechanical stress can also occur in diagnostic ultrasound settings, where the stable oscillation of microbubbles is detected in contrast-specific imaging. Furthermore, destruction-replenishment methods, including violent microbubble destruction, are often used to assess tissue perfusion [19] or detect specific receptors during molecular ultrasound via the destruction of specifically bound microbubbles [20]. Although standard clinical contrast-enhanced ultrasound protocols are considered comparatively safe diagnostic interventions, microvascular injury in the rat mesentery [21] and glomerular capillary hemorrhage in rat kidneys [22] were reported as a consequence of oscillation or violent collapse of microbubbles in small vessels.

**Fig. 2.**
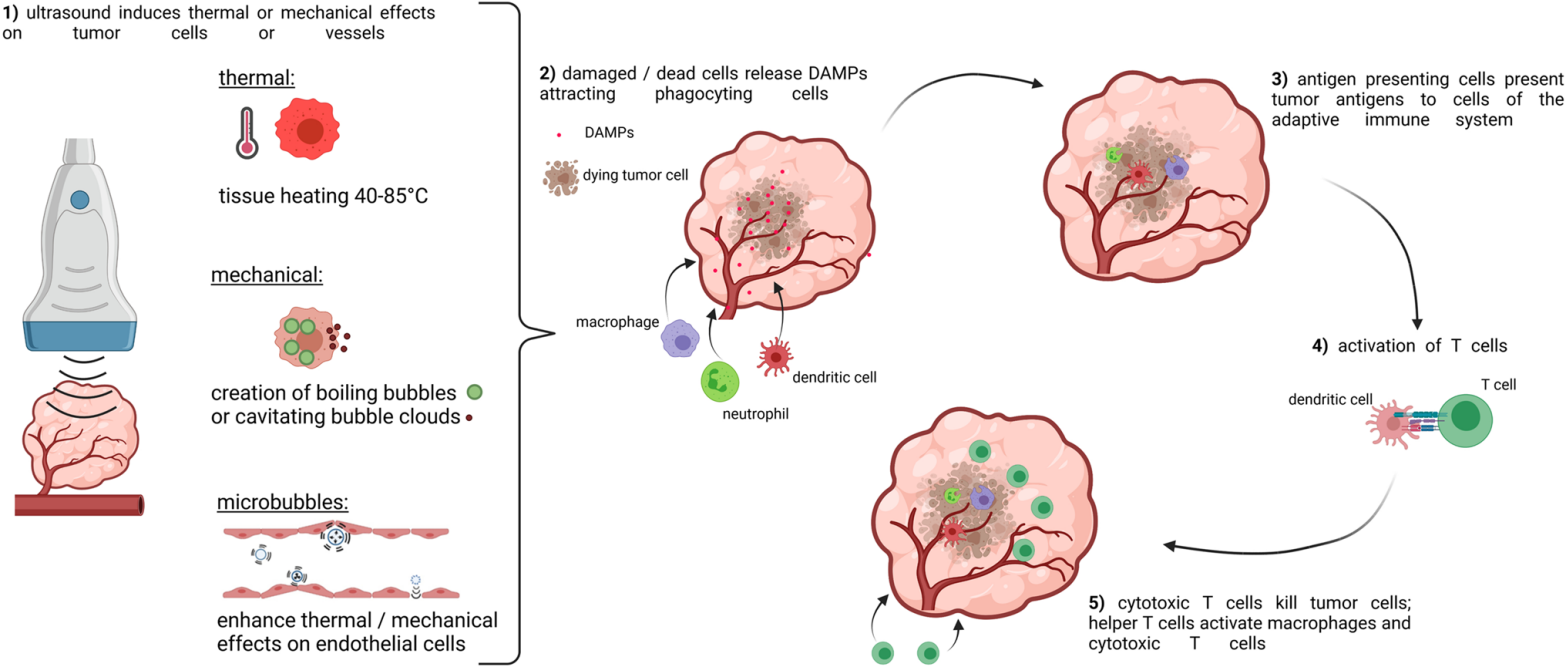
Immune effects in tumors induced by ultrasound interventions. Ultrasound can induce thermal or mechanical effects. These effects can be enhanced on endothelial cells by ultrasound contrast agents (gas-filled microbubbles) (**1**). Independent of the underlying mechanism, ultrasound can lead to damage or destruction of tumor cells. These cells release danger-associated molecular patterns (DAMPs), which recruit phagocyting cells (e.g., macrophages, neutrophils, dendritic cells) (**2**). Antigen-presenting cells, e.g., dendritic cells, present tumor antigens to the adaptive immune system (e.g., T cells) (**3**, **4**). Cytotoxic and T helper cells infiltrate the tumor tissue and activate macrophages or other cytotoxic T cells (**5**). (created with BioRender.com).

Independent of the underlying mechanism, the ultrasound-induced immune response in tumors originates from cell stress or cell death. In detail, cells of the innate immune system (e.g., dendritic cells, neutrophils, macrophages) are activated via danger-associated molecular patterns (DAMPs) released from damaged or dying cells [23]. After endocytosis, tumor antigens are presented on the surface of antigen-presenting cells, like dendritic cells. These can be recognized by cells of the adaptive immune system, leading to differentiation and activation of T cells (cytotoxic T cells or T helper cells). Depending on the type of T cell, the immune response can be further enhanced or attenuated [24]. Next to provoking an immune reaction, ultrasound can also alter this reaction in different ways. For example, high temperatures present during tumor ablation can denature proteins (e.g., tumor antigens) so that they can no longer be recognized by immune cells [2]. Furthermore, the tumor vasculature can be altered either by ultrasound or indirectly via oscillating or violently collapsing microbubbles, leading to a vascular breakdown, preventing the migration of immune cells from the blood system, and inhibiting the immune reaction. On the other hand, oscillating microbubbles may also increase tumor vascularization and vessel permeability, enabling an increased infiltration of immune cells into the tumor [3] (Fig. 2).

Although the prediction of the immune response is difficult because of the multitude of parameters that can have an influence, this information would be important to refine specific ultrasound settings to trigger the best anti-tumor immune response but also to avoid immune effects in diagnostic applications. In addition, slight immune reactions possibly influence study outcomes when using ultrasound for therapy monitoring in preclinical drug discovery. This can lead to a false interpretation of data which can be a source for low reproducibility between experiments and finally impair clinical translation.

As a first step in creating a meaningful summary of the relevant literature, we present a new protocol for a systematic review to answer the following research question: Does ultrasound alter the immune reaction of peripheral solid tumors in humans and animals compared to control conditions without ultrasound? We registered the protocol in the international prospective register of systematic reviews PROSPERO (registration number CRD42021248743). Next, we tested the suitability of the protocol to detect and sort relevant literature on a defined subset of publications from a preliminary literature search. Finally, a scoping review of the selected literature on study characteristics, ultrasound parameters, and study outcomes was performed to assess the differences in study parameters between publications.

## Materials and Methods

### Development of the Systematic Review Protocol

We registered the systematic review protocol in PROSPERO (registration number CRD42021248743). The items covered by the protocol are described in more detail in the following paragraphs.

### Research Question (PICO), Search Terms, and Search Strategy

We formulated the research question following the PICO (population, intervention, comparison, outcome) format [25]: does ultrasound (intervention) alter the immune reaction (outcome) of peripheral solid tumors in humans and animals (population) compared to control conditions without ultrasound (comparison)?

Publications need to fulfill the following requirements for inclusion: Studies of all animals and humans, of both sexes, and any age with a solid peripheral tumor that can be visualized with ultrasound. Tumors of the central nervous system will be excluded, as the immune system in the brain is different from the rest of the body [26]. Furthermore, cancers that do not develop a solid tumor (e.g., leukemia) or tumors that cannot be visualized by ultrasound (e.g., bone cancer) will be excluded. Any type of ultrasound, either diagnostic or therapeutic, will be another selection requirement. We do not restrict our search to specific outcomes a priori and analyze all information on immune parameters in ultrasound treated and untreated animals and humans with the specified tumors.

### Study Identification

The search strategy will be designed according to M. Leenaars et al. [27] and performed on different scientific databases: PubMed, EMBASE, and Web of Science. We aim to identify all relevant publications on ultrasound-induced immune effects in solid peripheral tumors. Therefore, the search strategy consists of four search strings: (1) all animal species and humans, (2) all cancer types of interest, (3) any ultrasound application, and (4) immune-related parameters.

We will use the SYRCLE filter [28] to identify animal studies. Human studies will be identified with a previously prepared search filter [29]. We will develop new custom search strings for the other topics comprising thesauri terms (Medical Subject Headings (MeSH) for PubMed or Emtree subject headings for Embase) and title-abstract-keyword terms. These terms are combined with the Boolean operator “OR” within search strings to identify publications with any of the defined terms. The four search strings mentioned in the previous paragraph will be combined with the Boolean operator “AND” to guarantee that at least one defined term of each search string is present in the title or abstract of the identified publication. The current search string to identify the relevant literature in PubMed can be found in supplementary Table S1.

### Study Selection

After merging all search results and removing duplicate publications, the study selection will be conducted in two phases. First, two out of three reviewers will perform a title/abstract screening using the systematic review software Rayyan [30]. Publications of any language and any date will be considered if they fulfill the inclusion criteria listed in Table 1. Conflicting results between reviewers will be discussed with an independent person. If a publication cannot be judged by its title/abstract because of insufficient or unclear information, it will be included for the next selection phase.

**Table 1.** Criteria to screen full-text articles for eligibility for data extraction sorted according to their prioritization for the full-text screening phase

	**Inclusion criteria**	**Exclusion criteria**
1. Publication type	Full, peer-reviewed publication of a primary study (we plan to analyze methodological details—therefore, we need a full methodological description)	Other publication formats (e.g., pre-prints, conference abstracts, reviews without new data)
2. Type of animals/population	All animals, including humans with a solid peripheral tumor that can be visualized by ultrasound	No *in vivo* application No solid tumor Tumor of the central nervous system The tumor cannot be visualized by ultrasound
3. Type of intervention	Ultrasound performed on tumor	No ultrasound was performed Ultrasound performed unrelated to tumor
4. Outcome measures	Immune system measures reported	Immune system measures are not reported
5. Study design	Between-subject comparison: Assessment of outcome of interest in animals is only possible by histological analysis and can only be compared between different subjects	Within-subject comparison with baseline only
6. Comparator	Any control with a tumor but without ultrasound	Other control (e.g., no tumor, within-subject control, control with ultrasound)

In the second selection phase, two out of three reviewers will perform a comprehensive full-text screening of the remaining publications for eligibility for data extraction. In this phase, the exclusion criteria are prioritized as described in Table 1, and the highest-ranking reason for exclusion will be recorded. Again, conflicting results will be discussed with an independent reviewer.

### Data Extraction

After completing both screening phases, all remaining publications will be divided between two reviewers for data extraction. A third reviewer will verify the accuracy of extracted data for a subset of included publications. The extraction contains relevant information to identify each study (e.g., author names, year of publication, study title, journal, page numbers), study design characteristics (e.g., experimental groups, number of animals per group), ultrasound settings (e.g., wavelength, pressure, energy, timing, duration, type, and concentration of contrast agent) and all immune-related outcomes (e.g., percentage/number of dendritic cells, T cells, macrophages) in tumor or blood. Data presented in the text or tables will be preferentially recorded for quantitative data. If this is not possible, a digital ruler (e.g., WebPlotDigitizer [31]) will be used to extract data from graphs. If data cannot be obtained by these methods or other essential information is missing, the publications’ authors will be contacted via email.

### Risk of Bias Analysis

The risk of bias (RoB) of all included publications will be evaluated using SYRCLE's RoB tool [13] for assessing the risk of bias in animal studies or the Cochrane RoB2 tool for randomized clinical trials [32]. Both RoB tools are designed to detect different types of bias, e.g., selection bias, performance bias, detection bias, attrition bias, and reporting bias [33]. The reporting bias includes assessing selective outcome reporting and will only be analyzed in animal studies if the study protocol is available in an animal study registry. Two reviewers will independently rate all items for each included publication. Publications will be rated to have a “low risk” if methods used to minimize the RoB are described adequately. If methods are described that do not meet the criteria to reduce the RoB properly, then the specific item will be rated as a “high risk”. An insufficient reporting of methods will be rated as an “unclear risk” [13].

### Data Synthesis and Meta-analyses

The number of identified publications in each screening phase and reasons for exclusion will be summarized in a flowchart according to the PRISMA statement [34]. Extracted data will be presented in tabular form. All immune-related outcomes reported in tumor or blood will be narratively summarized, and ranges and median values will be reported for immune cell counts described in multiple studies. A meta-analysis will be performed if more than five publications report the same cell type. If many outcomes are reported by five or more publications, the total number of meta-analyses will be restricted to the three most reported outcomes for tumors and the three most reported outcomes for blood.

For the meta-analysis, a random-effects model is used to compare effect sizes even with substantial differences in species, tumor models, and ultrasound settings among the included publications. The effect sizes of all included publications are considered as a random distribution of all possible effects. The random-effects model is used to calculate the mean effect size of all included publications. In addition, the heterogeneity of effect sizes between included publications is assessed using the *I*^2^ statistic, ranging from 0 to 100 %, with values below 25 % considered low heterogeneity, indicating that effect sizes are very similar and not influenced by other study characteristics. Values above 75 % indicate high heterogeneity between effect sizes, which means that the effect is influenced by an unknown factor that warrants further investigation (e.g., subgroup analysis or meta-regression) [35]. The following parameters can be investigated as sources of heterogeneity in subgroup analyses: ultrasound settings, additional treatment, tumor type, animal species, animal strain, age, or time of data collection. The results of the meta-analysis are presented in the form of a forest plot.

Further necessary corrections for meta-analyses (e.g., for multiple testing and multiple uses of the same control groups) will be performed according to Borenstein et al. [35].

### Assessment of Small-Study Effects

In a last step, we will assess small-study effects, which describe the problem that small studies with larger effects seem more likely to be published than those with smaller or zero effects (also referred to as publication bias) [35]. Potential small-study effects will be visualized in a funnel plot for each meta-analysis with more than ten included publications. The effect size is plotted on the *X*-axis and the standard error on the *Y*-axis. Without small-study effects, the effect sizes of all publications will spread symmetrically around the mean. Quantitatively, this will be assessed with a trim-and-fill analysis. An asymmetry in the funnel plot will be compensated by removing (trimming) asymmetric values and re-adding them with a mirror image (filling). Comparing the trim-and-fill analysis’ result with the original mean effect size will show the potential impact of a small-study effect.

## Scoping Review

We conducted a scoping review for a defined subset of publications to provide an overview of the available literature regarding the potential number of publications and the expected differences in study characteristics between publications. The 100 newest and 100 oldest publications and 100 publications from the middle of the list (total *k* = 300) were selected, and the suitability of the systematic review protocol was tested on this subset. The title/abstract screening was performed by three reviewers using Rayyan [25]. Additionally, 20 publications from recent review articles [8, 9, 13] were added to the full-text screening. These 20 publications were also identified by our preliminary search strategy but were initially not added to the title/abstract screening as their date of publishing was not included in the selected subset. The full-text screening and data extraction were performed according to the methods described in the systematic review protocol.

## Results of the Scoping Review

Our preliminary search in PubMed retrieved *k* = 6532 potentially relevant publications. Next, the title/abstract screening, performed on a subset of 300 publications, resulted in 18 publications suitable for the full-text screening phase.

**Fig. 3.**
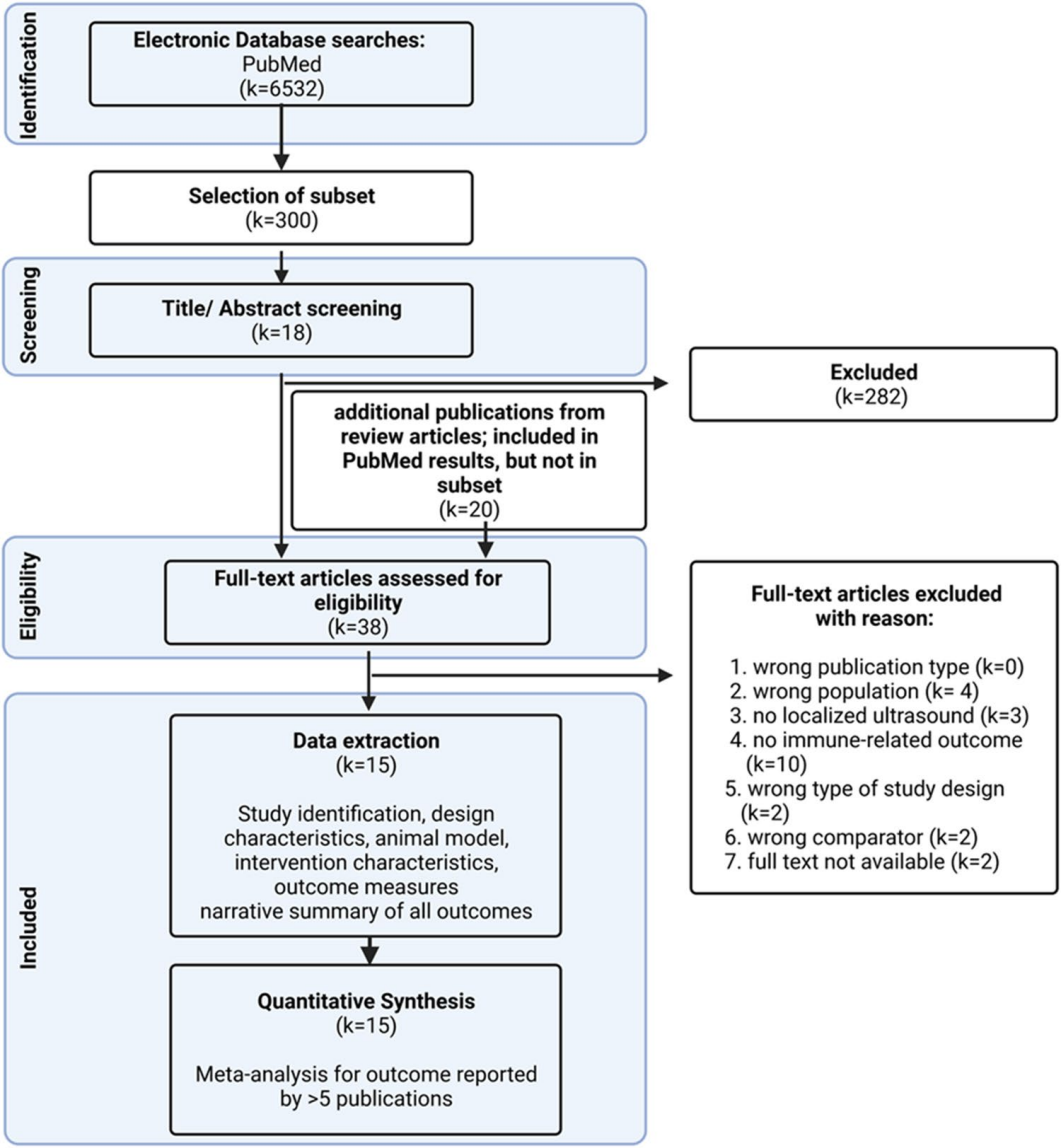
Flow chart of the scoping review. The number of publications included in the separate screening phases and the number and reason for excluding publications in the full-text screening phase are presented (created with BioRender.com).

Full-text screening of 38 publications (18 from title/abstract screening and 20 from recent review articles) resulted in 15 publications for data extraction. Seven of these 15 publications originate from the subset included in the title/abstract screening (2 % of all publications included in the title/abstract screening) and 8 from recent review articles. Twenty-three publications were excluded in the full-screening phase because of the following reasons: a lack in reporting an immune-related outcome (*k* = 10), using a wrong population (*k* = 4), wrong intervention (*k* = 3), wrong comparator (*k* = 2), not including a proper study design (*k* = 2), or the full texts were not available (*k* = 2). An overview of the included publications in the different screening phases can be seen in Fig. 3.

### Data Extraction and Study Characteristics

**Fig. 4.**
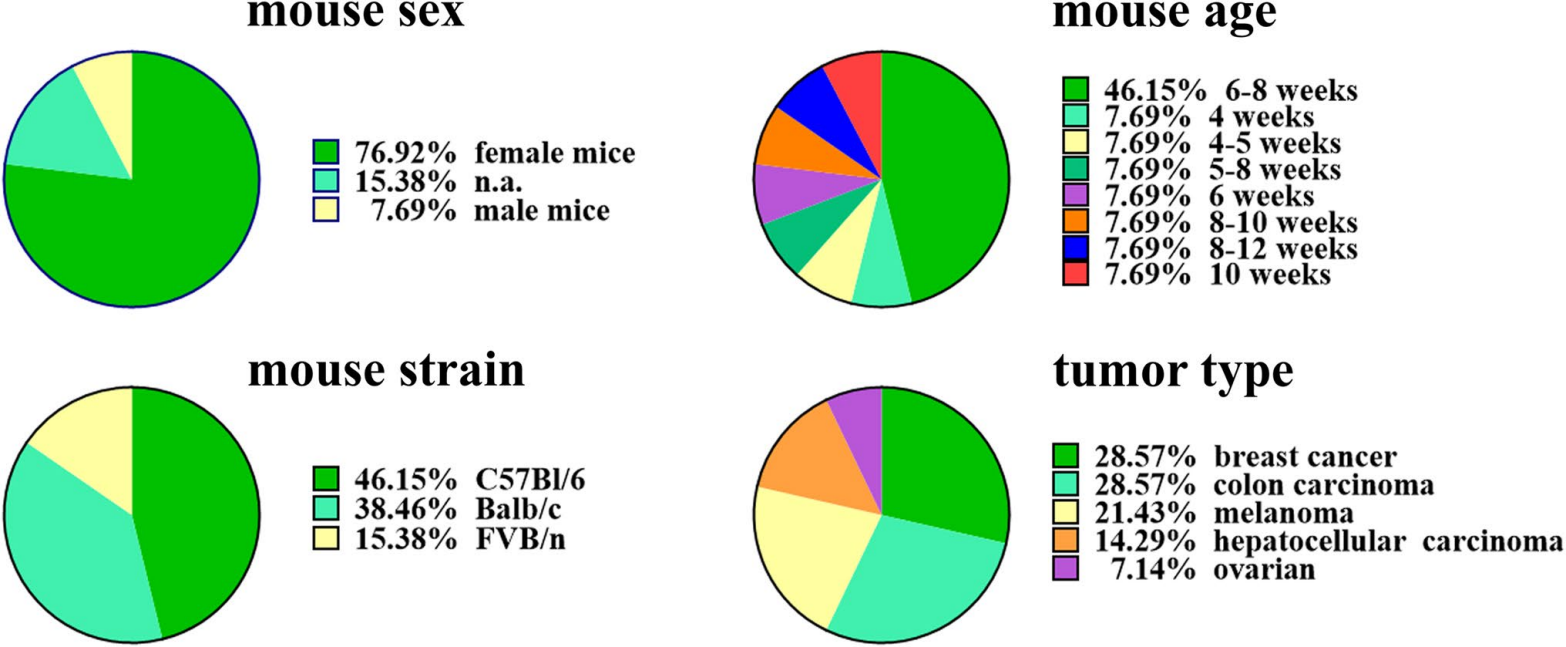
Differences in study characteristics between publications. Pie charts highlight the study characteristics of mouse sex, mouse age, mouse strain, and tumor type as described in the small subset of publications included in the scoping review. The characteristics show large differences between publications.

First, we extracted all study characteristics to get an impression of the differences in the experimental setup between publications. The majority (13/15) of included publications used mice, whereas two showed results of female human breast cancer patients aged 46–47 years [36, 37]. Regarding publications performed in mice, female mice were used in 76.92 % (11/13) of publications [38–47]. Furthermore, one publication was performed with male mice (7.69 %) [48], and two publications did not report the mice’s sex (15.38 %) [49, 50] (Fig. 3A). The mouse age could be identified as another highly divergent factor between publications, with 46.15 % of publications using mice aged 6–8 weeks. In contrast, the other publications used animals between 4 and 10 weeks of age (Fig. 4A). Finally, the mouse strains (C57Bl/6, Balb/c, and FVB/n; Fig. 4A) and tumor types (melanoma, colon carcinoma, breast cancer, hepatocellular carcinoma, and ovarian cancer; Fig. 4A) varied in the small subset of publications analyzed in this scoping review.

Furthermore, the ultrasound settings differed substantially between publications (e.g., frequency 0.5–9.5 MHz, acoustic power 3–450 W, power density 0.0001–15,000 W/cm^2^, duty cycle 0.5–75 %), although all included publications used therapeutic ultrasound applications.

A summary of study designs, ultrasound settings, and reported outcomes of all included publications can be found in supplementary Table S2.

### Overview of Immune-Related Outcomes

Most publications report results on CD8-positive T cells in tumors (12/15), followed by CD4-positive T cells (10/15). Information on tumor macrophages could be found in six and information on dendritic cells in five publications. Furthermore, tumor-associated natural killer cells, B cells, and CD4 Foxp3-positive T cells were reported by two and myeloid-derived suppressor cells, granulocytes, mast cells, and leukocytes by one publication. Finally, two publications report various inflammatory cytokines, chemokines, and DAMPs in tumors and blood. An overview of all outcomes can be found in supplementary Table S2.

## Discussion

The number of publications on new cancer treatments is steadily increasing; however, their success rates in clinical applications are still considerably low. According to Begley and Ellis, the poor reproducibility and robustness of published preclinical data contribute to this limitation [51]. This situation could be improved by comprehensively analyzing the large amount of published literature to generate more robust and evidence-based conclusions and to elucidate mechanistic connections. In this regard, systematic reviews and meta-analyses follow explicit, pre-defined methods to gather all available empirical research [4]. In the end, these data can help identify knowledge gaps that could be answered by conducting further (preclinical) experiments, help to prevent unnecessary duplications of experiments, and they can help to refine the design and conduct of future experiments [11]. Therefore, systematic reviews can increase the robustness of conclusions to validate or reject scientific hypotheses. Furthermore, they substantially contribute to the concept of replacing, reducing, and refining the use of animals in preclinical research, which was first introduced by Russel and Burch [52].

Using a published and pre-registered protocol to perform the systematic review and meta-analyses minimizes the risk of introducing a methodological bias, as the protocol cannot easily be adapted retrospectively to suit the authors’ preference [6]. Therefore, we here present a systematic review protocol to answer the question: does ultrasound alter the immune reaction of peripheral solid tumors in humans and animals compared to control conditions without ultrasound? Furthermore, we tested the suitability of our protocol on a subset of literature and present a scoping review of the extracted data in this manuscript.

The criteria defined in the protocol enable us to identify and sort relevant publications for data extraction. We analyzed a subset of publications as a scoping review, which is defined as a preliminary exploratory assessment of the available literature on a given topic [4] to estimate the number of available publications and assess the differences in study characteristics between publications. Here, we found that 2 % of publications from the preliminary search were eligible for data extraction. The estimate of 2 % of publications means that if we include all results from our literature search in one database (*k* = 6532), we will have around 130 publications eligible for data extraction in the systematic review, which will be enough to perform subgroup analyses and identify potential publication bias in most cases.

The extraction of study characteristics showed large differences in mouse strains, sex, tumor types, and age. Age is a crucial aspect in investigating immune-related parameters, as the immune system naturally changes over time. Therefore, interventions performed in juvenile animals can lead to different results than those performed in elderly animals. In this small subset, the mouse age varied between 4 and 10 weeks, which can be critical, as, e.g., the number of white blood cells in mice increases between 2 and 9 weeks of age [53]. In contrast to preclinical publications reporting results from young mice, both clinical publications present results from middle-aged women (46–47 years), corresponding to mice aged 10–14 months [54]. Starting in the late thirties, the number of T cells in the human blood already starts to decline, whereas the number of monocytes increases [55]. These aspects show that age is an important source of heterogeneity for immune-related outcomes.

The extraction of study outcomes showed that various cells contributing to the innate and the adaptive immune reaction were investigated after ultrasound treatment. Therefore, changes in both stages of the immune reaction can be assessed by the systematic review.

In conclusion, we present a protocol to find and sort relevant literature and perform a systematic review to answer our research question. A scoping review on a subset of the literature revealed a large difference between study characteristics, ultrasound settings, and reported outcomes, demonstrating the need to include a comprehensive sample of publications to generate a clear conclusion from the data. In the future, the results from the complete systematic review and meta-analysis may then assist in unraveling the influence of ultrasound on the tumors’ immune response. In addition, these data can help to refine therapeutic ultrasound applications or highlight the risk for inducing an experimental bias in diagnostic ultrasound settings.

## Supplementary Information

Below is the link to the electronic supplementary material.

Supplementary file1 (DOCX 24 kb)
