# Supplementary material for: Development of a Systematic Review Protocol and a Scoping Review of Ultrasound-Induced Immune Effects in Peripheral Tumors
Source: Mol Imaging Biol. 2021 Nov 29;24(2):288–97. doi: 10.1007/s11307-021-01686-x (PMC8983530; doi:10.1007/s11307-021-01686-x)
Supplement: Supplementary file 1 — Supplementary file1 (DOCX 24 kb) [file 11307_2021_1686_MOESM1_ESM.docx]

**Supplementary Material**

**Development of a systematic review protocol and a scoping review of ultrasound-induced immune effects in peripheral tumors**

Anne Rix*, Renée Girbig, Céline Porte, Wiltrud Lederle, Cathalijn Leenaars, Fabian Kiessling*

Supplementary table S1: complete search strategy created to identify all relevant literature in Pubmed

| **Search string element** | **Search string** |
| --- | --- |
| Humans / animals | Human search string adapted from [24]: clinical study [pt] OR clinical trial [MeSH] OR clinical trial [tiab] OR intervention study [tiab] OR controlled clinical trial [MeSH] OR first in man [tiab] OR proof of concept [tiab] OR randomized controlled trial [pt] OR controlled clinical trial [pt] OR randomized [tiab] OR placebo [tiab] OR randomly [tiab] OR trial [tiab] OR groups [tiab] OR patient [tiab] OR patients [tiab]  to identify all animals, we used the search string as described in van der Mierden et al 2021 Laboratory Animals |
| Tumor | “neoplasms”[MeSH Terms:noexp] OR “neoplasms, complex and mixed”[MeSH Terms:noexp] OR “neoplasms, connective and soft tissue”[MeSH Terms:noexp] OR “neoplasms, germ cell and embryonal”[MeSH Terms:noexp] OR “carcinoma, adenosquamous”[MeSH] OR “carcinosarcoma”[MeSH] OR “hepatoblastoma”[MeSH] OR “mesenchymoma”[MeSH] OR “mixed tumor, malignant”[MeSH] OR “mixed tumor, mesodermal”[MeSH] OR “mixed tumor, mullerian”[MeSH] OR “nephroma, mesoblastic”[MeSH] OR “pulmonary”[MesH] OR “blastoma”[MeSH] OR “rhabdoid tumor”[MeSH] OR “sarcoma, endometrial stromal”[MeSH] OR “thymoma”[MeSH] OR “wilms tumor”[MeSH] OR “liposarcoma”[MeSH] OR “chondrosarcoma”[MeSH] OR “endometrial stromal tumors”[MeSH] OR “gastrointestinal stromal tumors”[MeSH] OR “fibrosarcoma”[MesH] OR “sarcoma, clear cell”[MeSH] OR “sarcoma, small cell”[MeSH] OR “sarcoma, synovial”[MeSH] OR “leiomyosarcoma”[MeSH] OR “myosarcoma”[MeSH] OR “sarcoma, alveolar soft part”[MeSH] OR “smooth muscle tumor”[MeSH] OR adenosarcoma[MeSH] OR “desmoplastic small round cell tumor”[MeSH] OR hemangiosarcoma[MeSH] OR lymphangiosarcoma[MeSH] OR myxosarcoma[MeSH] OR “sarcoma, experimental”[MeSH] OR “sarcoma, myeloid”[MeSH] OR carcinoma[MeSH] OR “neoplasms, basal cell”[MeSH] OR “carcinoma, medullary”[MeSH] OR “pancreatic intraductal neoplasms”[MeSH] OR mesothelioma[MeSH] OR retinoblastoma[MeSH] OR “neoplasms, gonadal tissue”[MeSH] OR craniopharyngioma[MeSH] OR apudoma[MeSH] OR “neoplasms, squamous cell”[MeSH] OR melanoma[MeSH] OR “abdominal neoplasms”[MeSH] OR “anal gland neoplasms”[MeSH] OR “breast neoplasms”[MeSH] OR “digestive system neoplasms”[MeSH] OR “endocrine gland neoplasms”[MeSH] OR “head and neck neoplasms”[MeSH] OR “mammary neoplasms, animal”[MeSH] OR “pelvic neoplasms”[MeSH] OR “soft tissue neoplasms”[MeSH] OR “splenic neoplasms”[MeSH] OR “thoracic neoplasms”[MeSH] OR “urogenital neoplasms”[MeSH] OR “neoplasms, experimental”[MeSH] OR “neoplasms, hormone-dependent”[MeSH] OR “neoplasms, multiple primary”[MeSH] OR neoplasm[tiab] OR neoplasias [tiab] OR neoplasia [tiab] OR tumor[tiab] OR tumour[tiab] OR tumors[tiab] OR tumours[tiab] OR cancer*[tiab] OR malignan*[tiab] OR lymphangiosarcoma*[tiab] OR hemangiopericytoma*[tiab] OR hemangiosarcoma*[tiab] OR melanoma*[tiab] OR choriocarcinoma*[tiab] OR adenocarcinoma*[tiab] OR adenocarcinoma*[tiab] OR sarcoma*[tiab] |
| ultrasound | elasticity imaging techniques[MeSH] OR endosonography[MeSH] OR microscopy, acoustic[MeSH] OR Ultrasonography, Doppler, Duplex[MeSH] OR Ultrasonography, Doppler, Pulsed[MeSH] OR Ultrasonography, Mammary[MeSH] OR ultrasonic waves[MeSH] OR high-intensity focused ultrasound ablation[MeSH] OR echogr*[tiab] OR ultraso*[tiab] OR sonogr*[tiab] OR echoscopy[tiab] OR echosound[tiab] OR “b mode”[tiab] OR CEUS[tiab] OR Doppler[tiab] OR echomammography[tiab] OR elastography[tiab] OR LIFU[tiab] OR LIFUS[tiab] OR LIPUS[tiab] OR MRgFUS[tiab] OR pHIFU[tiab] OR HIFU[tiab] OR sonodynamic[tiab] OR UTMD[tiab] OR microbubble[tiab] OR microbubbles[tiab] OR bubble[tiab] OR bubbles[tiab] |
| Immune system | “immune system”[MeSH] OR “macrophages”[MeSH] OR “monocytes”[MeSH] OR “leukocytes”[MeSH] OR “leukocyte count”[MeSH] OR “heat-shock proteins”[MeSH] OR interleukins[MeSH] OR interferons[MeSH] OR alarmins[MeSH] OR tumor necrosis factor-alpha[MeSH] OR (inflamma*[tiab] OR immuni*[tiab] OR immunology[tiab] OR immunotherap*[tiab] OR “immune system*”[tiab] OR immune[tiab] OR immunological[tiab] OR immune[tiab] OR immunogenicity[tiab] OR immunosuppressive[tiab] OR immunotoxicity[tiab] OR macrophage*[tiab] OR lymphocyte*[tiab] OR “B cell*”[tiab] OR “dendritic cell*”[tiab] OR “veiled cell*”[tiab] OR “interdigitating cell*”[tiab] OR leukocyte*[tiab] OR “white blood cell*”[tiab] OR “white blood corpuscle*”[tiab] OR granulocyte*[tiab] OR basophil*[tiab] OR eosinophil*[tiab] OR neutrophil*[tiab] OR T cell*[tiab] OR NK cell*[tiab] OR "natural killer cell*”[tiab] OR monocyte*[tiab] OR “LE cell*”[tiab] OR “inflammatory cell*”[tiab] OR “Kupffer cell*”[tiab] OR microglia*[tiab] OR interleukin*[tiab] OR IL[tiab] OR interferon*[tiab] OR IFN[tiab] OR “tumor necrosis factor”[tiab] OR TNF[tiab] OR “heat-shock protein*”[tiab] OR HSP[tiab] OR DAMP[tiab] OR “danger associated molecular pattern”[tiab] OR “danger-associated molecular pattern”[tiab] OR “antigen-presenting cell”[tiab] OR “antigen-presenting cells”[tiab] OR “antigen presenting cell”[tiab] OR “antigen presenting cells”[tiab] |

| **Supplementary table S2:** Narrative summary of study design, ultrasound parameters and outcomes extracted from all publication | | | | |
| --- | --- | --- | --- | --- |
|  | **Species and tumor type** | **Experimental groups**  **(subjects / group)** | **Ultrasound parameters and additional treatment** | **Immune parameters and time point of assessment** |
| Bandyopadhyay 2016 | mouse C57Bl/6  6-8 weeks,  **tumor:**  B16-F1  melanoma | 1. control (3-4) 2. LOFU (3-4) 3. HIFU (3-4) | 1 MHz  LOFU (1x):  acoustic power 3W, peak negative pressure: 2.93 MPa / 3.81 MPa, power density 550 W/cm^2^, 1.5s/ spot, total 5 min  HIFU (1x):  pressure: 5.42 MPa; duty cycle 75%; acoustic power 12.5 W; 4s / spot | 36 h after treatment:  leukocytes, CD4^+^ T cells, CD8^+^ T cells, CD4+ Foxp3+ T cells; CD69 expression on CD4^+^ and CD8^+^ |
| Bulner 2018 | mouse Balb/c  female, 8-12 weeks  **tumor:**  CT26  colon carcinoma | 1. MB (6) 2. MB+LOFU (5) | 1x: 1 MHz, 1.65 MPa peak negative pressure; 50x 0.1ms pulses spaced 1ms apart, repeated at 20s intervals; 2 min total  additional:  9.6 x 10^8^ octafluoropropane MBs/kg bolus 10s prior to US | d3 & d7 after treatment:  leukocytes, CD4^+^ T cells, CD8^+^ T cells; ratio CD4^+^/CD8^+^ |
| Chavez 2018 | mouse FVB/n  female, 4-5 weeks  **tumor:**  NDL  breast cancer | 1. IT (8) 2. FUS + IT (8) | 1x: 3 MHz, thermal ablation above 60°C  additional:  anti PD-1 (200µg, i.p.) day: 21, 28, 34 after implantation  CpG (100µg i.t.) day: 21, 24, 28, 31 after implantation | d7 after treatment  T cells, CD4^+^ T cells, CD8^+^ T cells, activated CD8^+^ T cells; macrophages; dendritic cells |
| Chen 2021 | mouse Balb/c  female, 6 weeks  **tumor:**  4T1  breast cancer | 1. PBS (5) 2. PBS + US (5) | 1x: 1 MHz, 50% duty cycle, power density 2 W/cm^2^, 5 min | 2 days after last treatment:  CD4^+^ T cells, CD8^+^ T cells, natural killer cells; macrophages; dendritic cells |
| Ektate 2018 | mouse Balb/c  female, 10 weeks  **tumor:**  C26  colon carcinoma | 1. control (6) 2. HIFU (6) | 1x: 1 MHz, 35% duty cycle, PRF 5 Hz, acoustic power 6 W, 60s / point; total ~ 3 min | 4 days after treatment:  T cells, CD4^+^ T cells, CD8^+^ T cells, activated CD4^+^ and CD8^+^ T cells; macrophages; dendritic cells; granulocytes |
| Fite 2021 | mouse FVB/n  female, 4 weeks  **tumor:**  NDL  breast cancer | 1. control (6) 2. thermal ablation (4) 3. mechanical ablation (5) | thermal ablation 1x: 3 MHz, peak negative pressure 3.1 MPa, continuous wave until targeted volume reached 60°C  mechanical ablation (1x): 3 MHz, peak negative pressure 16.9 MPa, 0.5% duty cycle, 5ms / spot | mechanical: 72h after ablation/ thermal: 1 week after ablation:  macrophages; dendritic cells; MDSCs, various inflammatory cytokines and leukocyte signaling chemokines |
| Lin 2021 | mouse C57Bl/6  female, 6-8 weeks  **tumor:**  Hepa1-6  hepatocellular carcinoma | 1. PBS (6) 2. PBS+US (6) | 1x:  1 MHz, 50% duty cycle, power density 2 W/cm^2^, 7 min  additional:  PBS injection intra tumoral one day before US | 1 day after treatment:  CD8^+^ T cells, dendritic cells |
| Liu 2010 | mouse C57Bl/6  female, 5-8 weeks  **tumor:**  B16  melanoma  MC-38  colon carcinoma | 1. control (6) 2. sparse scan HIFU (6) 3. dense scan HIFU (6) | mechanical ablation (1x):  3.3 MHz, peak negative pressure 7.2 MPa, 4s / spot; sparse scan: 2mm between spots; dense scan: 1mm between spots | 1 day after treatment:  dendritic cells |
| Liu 2012 | mouse  Balb/cByJNarl  8-10 weeks  **tumor:**  CT26  colon carcinoma | 1. control 2. 0.6 MPa 3. 1.4 MPa | mechanical stress:  0.5 MHz, peak negative pressure 0.6 MPa/ 1.4 MPa, PRF 1 Hz, acoustic power 5 W / 30 W, 20s / spot = 180 -240 s total  additional:  0.1 ml/kg SonoVue^®^ in 0.2ml saline + 0.2ml heparin | d18 after treatment / additionally 1.4 MPa d 1, 3, 18 after treatment:  CD4^+^ T cells, CD8^+^ T cells, CD4+ Foxp3+ T cells, mast cells |
| Lu 2009 | human  female, 46-47 years  **tumor:**  breast cancer | 1. control (25) 2. HIFU (23) | 1.6 MHz, 5000 – 15000 W/cm^2^, 45min – 2.5h | 7-14 days after treatment:  T cells, CD4^+^ T cells, CD8^+^ T cells, B-lymphocytes, natural killer cells |
| Ran 2015 | mouse C57Bl/6  female, 6-8 weeks  **tumor:**  H22  hepatocellular carcinoma | 1. sham HIFU (30) 2. HIFU (30) | 9.5 MHz, acoustic power 5 W, 180 – 240s | 14 days after treatment:  T cells, CD4^+^ T cells, CD8^+^ T cells,  ratio CD4^+^ / CD8^+^ |
| Si 2021 | mouse Balb/c  female, 6-8 weeks  **tumor:**  4T1  breast cancer | 1. PBS (6) 2. LOFU (6) | 6x: power density 0.0001 W/cm^2^, 2 min  Day: 10, 14, 17, 21, 24 | 4 days after last treatment:  CD4^+^ T cells, CD8^+^ T cells, IFN-γ, CRT, ATP |
| Singh 2021 | mouse C57Bl/6  male, 6-8 weeks  **Tumor:**  B16F10  melanoma | 1. control (3-5) 2. boiling histotripsy (3-5) | 1x: 1.5 MHz, peak negative pressure ~ 16 MPa, 1% duty cycle, PRF 1 Hz, acoustic power 450 W, 10s / spot | 1 week after treatment:  leukocytes, T cells, CD8^+^ T cells, activated CD8^+^ T cells |
| Xu 2009 | human  female, 46-47 years  **Tumor:**  breast cancer | 1. control 2. HIFU | 1x: 1.6 MHz, 5000 – 15000 W/cm^2^, 45min – 2.5h | 7-14 days after treatment:  macrophages, B-lymphocytes |
| Zheng 2021 | mouse C57Bl/6  female 6-8 weeks  **tumor:**  ID8  ovarian cancer | 1. OIX-NPs 2. OIX-NPS + PSDT | mechanical stress (4x):  power density 1 W/cm^2^, 1 min  additional:  oxaliplatin NP 2mg/kg, 30µl i.v., 4x every 5 days  photoacoustic imaging (together with US) 808 nm; 1.5W/cm^2^, 5min | 24 h after last treatment:  CD4^+^ T cells, CD8^+^ T cells, CRT, HMGB1, IFN-γ |
